# THE RELEVANCE OF COGNITIVE ABILITIES IN FRAUD DETECTION

**DOI:** 10.1093/geroni/igad104.3659

**Published:** 2023-12-21

**Authors:** Shenghao Zhang, Neil Charness, Walter Boot

**Affiliations:** Florida State University, Tallahassee, Florida, United States; Florida State University, Tallahassee, Florida, United States; Florida State University, Tallahassee, Florida, United States

## Abstract

Some previous studies suggest that cognitive decline might be responsible for older adults being susceptible to phishing attempts and frauds, but results are not consistent. The current study utilized a structural equation modeling approach to examine the role of different cognitive abilities in fraud detection. We used data from the baseline of the Intervention Comparative Effectiveness for Adult Cognitive Training (ICE-ACT, NCT03141281) Trial. The sample included 230 cognitively normal community-dwelling older adults (Mean age = 72). Reasoning was measured by Raven’s Advanced Progressive Matrices and letter sets. Memory was measured by Hopkins Verbal Learning Test and Rey Auditory Verbal Learning Test. Processing speed was measured by Digit Symbol Substitution Test and Useful Field of View. Fraud detection was represented by d-prime score calculated from rating of fraudulence in 10 vignettes describing fraud and non-fraud scenarios. Latent factors were created for reasoning, memory, and processing speed. Results showed that reasoning, memory, and processing speed explained 14% of variance in fraud detection, however, none of those cognitive factors uniquely predicted fraud detection. A higher-order general cognitive factor indicated by the reasoning, memory, and processing speed factors significantly predicted fraud detection and explained 13 % of variance. These results showed that general cognitive abilities have a medium effect in fraud detection in cognitively normal community-dwelling older adults. Discussion will focus on implications of those results on training and interventions aiming to prevent fraud in older adults.

